# Entropy Balance in the Expanding Universe: A Novel Perspective

**DOI:** 10.3390/e21040406

**Published:** 2019-04-17

**Authors:** Arturo Tozzi, James F. Peters

**Affiliations:** 1 Center for Nonlinear Science, Department of Physics, University of North Texas, Denton, TX 76203, USA; 2 Department of Electrical and Computer Engineering, University of Manitoba, 75A Chancellor’s Circle, Winnipeg, MB R3T 5V6, Canada

**Keywords:** quantum vacuum, Bekenstein bound, cosmological constant, ergodicity

## Abstract

We describe cosmic expansion as correlated with the standpoints of local observers’ co-moving horizons. In keeping with relational quantum mechanics, which claims that quantum systems are only meaningful in the context of measurements, we suggest that information gets ergodically “diluted” in our isotropic and homogeneous expanding Universe, so that an observer detects just a limited amount of the total cosmic bits. The reduced bit perception is due the decreased density of information inside the expanding cosmic volume in which the observer resides. Further, we show that the second law of thermodynamics can be correlated with cosmic expansion through a relational mechanism, because the decrease in information detected by a local observer in an expanding Universe is concomitant with an increase in perceived cosmic thermodynamic entropy, via the Bekenstein bound and the Laudauer principle. Reversing the classical scheme from thermodynamic entropy to information, we suggest that the cosmological constant of the quantum vacuum, which is believed to provoke the current cosmic expansion, could be one of the sources of the perceived increases in thermodynamic entropy. We conclude that entropies, including the entangled entropy of the recently developed framework of quantum computational spacetime, might not describe independent properties, but rather relations among systems and observers.

## 1. Introduction

Relational properties among quantum systems are the most fundamental elements to construct quantum mechanics, instead of being independent properties [1,2]. This strong claim, suggested by relational formulations of quantum mechanics, has been supported by recent papers, which state that the experimentally detected correlations in Bell tests strongly contradict the tenet of local realism, i.e., the properties of the physical world are independent of our observation of them [3]. According to relational formulations of quantum mechanics, features of quantum systems such as superposition and entanglement are manifested through the rules of counting the alternatives, without explicitly calling out the reference system. For example, wave function and reduced density matrix can be derived from the relational probability amplitude matrix [4].

Therefore, quantum mechanics has been reformulated as a theory that describes physical systems in terms of observer-dependent relational properties. This framework is inspired by the key idea behind special relativity, i.e., that the details of an observation depend on the reference frame of the observer. In this paper, we aim to use relational properties to describe two well-known physical phenomena, such as thermodynamic and information entropy, in terms of dependence from an observer embedded in their comoving cosmic horizon. In touch with relational quantum mechanics, our aim is to show that entropies do not describe independent properties, rather relations among systems and observers: this means that the complete description of information and thermodynamic entropies is only meaningful in the context of measurements performed by anobserver. Further, we show how this relational framework might be extended to also encompass the recently-developed theory of quantum computational spacetime and related entanglement entropy [5].

## 2. The Role of Entropies

### 2.1. Information

Information is a measurable physical quantity that, according to many scholars, might stand for the most general paradigm to investigate cosmological, physical and biological systems. It has been claimed that the physical world is made up of information itself [6], so that our Universe is assessable in pure terms of information. The idea that information is the fundamental physical quantity dates back to F.W. Kantor [7].By then, different information-related perspectives have been developed, from the hypothesis that the Universe is a giant digital computer [8,9], to the suggested link among information theory, statistical thermodynamics and the probabilistic nature of quantum mechanics [10,11,12,13], from computational loop quantum gravity [14] to connections between information and the Bekenstein–Hawking entropy [15,16].Therefore, information sits at the core of physics, leading to the proposal of the “it from bit” dogma [17]: every field or particle exists because of its observation. In our Universe, information, as well as energy, cannot be created or destroyed, i.e., the total number of cosmic bits must be kept constant. The conservation of information is derived from quantum field theory, via the quantum Liouville theorem [18].Indeed, quantum field theory (and information conservation) works both forward and backward in time. The probabilistic combination of pure states keeps the same set of probabilities. In other words, because time evolution is unitary, it is also reversible, i.e., no information can ever get lost: an observer is theoretically allowed, starting from any time-like slice, to run time backwards and compute what happened earlier. Therefore, the amount of information is invariant in our cosmos: the number of bits encompassed in the whole Universe cannot modify, even if the theory of “the world as a hologram” holds true [19,20].

One of the raised concerns against the paradigm of information preservations, i.e., the “black hole information paradox” related to the hypothetical loss of information inside black holes, has been solved: these puzzling cosmic bodies might release the “trapped” information through the Hawking radiation, until they evaporate [21,22,23]. It has been observed that the entropy of a black hole corresponds to the logarithm of a number of possible equally probable measurement choices *u* of the observer outside the event horizon. In effect, for entropy defined by

(1) $$H(u)=\int_{0}^{L}\rho \left(n,x\right)\ln\left(\rho \left(n,x\right)\right)dx,$$

we obtain a plot, representing symmetry of measurement choices of the observer inside the event horizon (amplitude going up one side the of a hill) and the observer outside the event horizon (amplitude going down the other side of a hill).

To avoid confusion or misconceptions, we need to make clear the difference between event and commoving horizons. The event horizon is the largest distance from which light emitted now can ever reach the observer in the future. The comoving horizon (also termed particle horizon) is the largest distance from which light could have reached the observer by a specific time: it stands for the space assessable by local observers [24]. The total amount of information encompassed in the event horizon is unvarying. Here we will show how the uncontroversial statement related to the conservation of cosmic information in the event horizon leads to unexpected consequences by the standpoint of a local observer embedded inside their own commoving cosmic horizon. Indeed, changes in entropy occur inside an asymptotically flat Friedmann–Robertson–Walker Universe when dust, radiation and black holes cross the cosmological commoving horizon and disappear [25].

### 2.2. Thermodynamic Entropy

In the previous paragraph, we stated that information is constant in our Universe. This may sound weird to anyone familiar with the second law of thermodynamics, which says that “every process occurring in nature proceeds in the sense in which the sum of the entropies of all bodies taking part in the process is increased” (Planck’s formulation). How can such opposite claims be consistent? The microscopic laws of physics are reversible: despite irreversibility that comes out due to coarse graining to a larger effective scale, the microscopic information does not get lost. Indeed, the entropy described by the second law is the sum of the entropies of many macroscopic local objects. Macroscopic physical systems, like the observable Universe, are not just regulated by stochastic variables and random fluctuations, but also by constraints given by the arrow of time.Despite the large number of different suggested scenarios and paradigms, the processes governing time constraints of physical and biological systems may be generalized, if we consider the universal principle of the second law of thermodynamics [26,27,28]. Therefore, the positive arrow of time observed in the macroscopic Universe (due to the time-reversal symmetry violation) is strictly correlated with the second law of thermodynamics. In thermodynamics, information I can be defined as the negation of thermodynamic entropy S [29]:

I = −S.(2)

In this account, a single bit of thermodynamic entropy stands for the distinction between two alternative states in a physical system. As a result, thermodynamic entropy of the Universe is proportional to the total number of distinguishable states encompassed in the cosmos: the higher the number of states, the higher the entropy. We will see in the sequel how thermodynamic entropy and information can be correlated, setting aside Shannon’s account [30].

## 3. Cosmic Expansion Comes into Play

The Universe is expanding. It has been hypothesized that our Cosmos arose from a perturbation in the quantum vacuum, when an inflationary mechanism, correlated with a false vacuum state, led to the production of cosmic matter and to the huge expansion that took place 1^−35^ s after the Big Bang [31]. Vacuum quantum fluctuations (dictated by the Heisenberg energy-time uncertainty principle) could have been able to cause, through an inflaton-based mechanism, the occurrence of the Big Bang, characterized by very high density and temperature state [32,33,34,35]. At the very beginning, 1^−43^ s after the Big Bang, our Universe was equipped with an energy of 10^19^ GeV and a temperature of 10^32^ K; its horizon was 10^−25^ cm large and the density 10^96^ kg/m^3^. By then, the temperature halved every double expansion. At 10^−36^ s, the energy lowered to 10^16^ GeV, and at 10^−32^ s the temperature decreased to 10^28^ K. The cosmic inflationary expansion at 10^−35^ s stands for the standard explanation for experimentally detected cosmic features such as isotropicity, homogeneity, symmetry and zero curvature. It is noteworthy that the Universe underwent a rapid expansion so that, from the above-mentioned horizon diameter of 10^−25^ at 10^−43^, it reached the size of about onemeter diameter at 10^−32^ s. Another gentler inflationary period started approximately 4.5 billion years ago [36]. Currently, 13.79 billion years after its birth, our Universe is still accelerating, slowly proceeding towards thermal death [37]. In our cosmic era, from our standpoint of local observers, the visible cosmological horizon is 10^29^ cm, the cosmic density is 10^−29^ gr/cm^3^, the matter corresponds to one atom/m^2^ and the space is expanding at a speed of about 67–74 km/s per megaParsec, with slightly different values according to different techniques [38].

How did (and still does) cosmic expansion occur? It has been correlated with the quantum vacuum, a material medium capable of polarization and equipped with its own electric permittivity, permeability and dielectric constant. The quantum vacuum is believed to display a negative pressure (an anti-gravitational force) that equals its energy density and causes the accelerated expansion of the current Universe. One of the feasible explanations of the quantum vacuum’santi-gravitational strength is the repulsive dark energy, which is correlated with the cosmological constant. The current consensus states that the cosmological constant gives rise to a negative pressure: indeed, the amount of energy in a container full of vacuum increases when the volume increases. The dark energy amount encompasses 73% of the whole Universe. This paradigm leads to a straightforward conclusion: due to the cosmic expansion, the density of “visible” matter and radiation is diluting inside observers’ commoving horizons. What about information? In the next paragraphs, we will elucidate how it is feasible to correlate cosmic expansion with information and thermodynamic entropy.

## 4. Linking Cosmic Expansion, Information and Thermodynamic Entropy

Cosmic expansion leads to an unexpected consequence: for a local observer enclosed in a given cosmological comoving horizon constrained by the light speed, the information density (bits/cosmic volume unity) is decreasing with time passing. This also means that observers perceive thermodynamic entropy as increasing. How is it possible? Here we illustrate the route that allows the correlation in a common theoretical framework of the relationships between thermodynamic entropy and information. The total entropy embedded inside the observer’s comoving horizon can be quantified through the Bekenstein bound, because the entropy must be finite in the sphere delimited by this horizon. The Bekenstein bound is an upper limit on the thermodynamic entropy S—or the information I, according to Shannon [30]—endowed in a space region equipped with a given amount of energy. In other words, the Bekenstein bound stands for the maximum quantity of information required to describe a physical system down to the quantum level. The universal form of the bound can be described as follows [39,40]:

(3) $$\mathrm{Ssys}=\zeta \frac{AEK}{\hbar c}$$

where S_sys_ is the cosmic thermodynamic entropy detectable by the observer, *A* is the area of the local observer’s horizon, *E* is the energy including matter (the total mass–energy of the Universe consists of about 10^69^ Joule), *ħ* is the reduced Planck constant, *c* is the speed of light, *k* is the Boltzmann constant, *ζ* is a factor such that 0 < *ζ* < 1.

Setting ζ to one in the case of the total S_sys_, we are allowed to quantify the thermodynamic information, by partitioning the factor into a relative information component (ζ_I_ = 1 − ζ_S_) and a relative entropy component (ζ_S_ = 1 − ζ_I_) [41]:

(4) $$I_{\mathrm{sys}}\;=\;\zeta I\frac{AEK}{\hbar c}=\;\left(1\;-\;\zeta S\right)\frac{AEK}{\hbar c}.$$

In the case of cosmic expansion, we achieve a decrease of information density in the space inside the observer’s comoving horizon.In other words, the number of detectable bits declines. This means that information exits from the observer’s comoving horizon, according to the formula:

(5) $$\Delta I_{\mathrm{sys}}\;=\;\frac{\Delta Esurr}{kT}=\;\Delta \zeta S$$

where *T* is the temperature. Note that temperature decreases with cosmic expansion, contributing to the cosmic budget of the thermodynamic entropy.

Here the Landauer principle comes into play: any logically irreversible manipulation of information, such as the erasure of a bit, must be accompanied by a corresponding entropyincrease in either the information-processing apparatus, or its environment [42]. The minimum possible amount of energy required to erase one bit of information is called the “Landauer limit”:

*kT*ln 2.

When one bit of logical information is lost, the amount of entropy generated is at least k ln 2, so that the energy which must eventually be emitted to the environment is *E* ≥ *kT* ln 2. To provide an example, at 20 °C, the Landauer limit represents an energy of approximately 0.0172 eV, or 2.75 zJ [43].

Due to our lack of knowledge of cosmic topology and shape, we cannot be sure that the system formed by the local observer’s event and comoving horizons are physically closed systems; in spite of this uncertainty, our general framework holds the same. Indeed, an increase in the number of physical states corresponding to each logical state means that, for an observer (a human “observer” embedded in its constrained comoving horizon) who is keeping track of the logical state of the system but not of its the physical state, the number of possible physical states has increased; in other words, entropy has increased from the standpoint of our observer.

In the whole Universe, the total expansion leaves the thermodynamic entropy of relativistic particles (such as photons, gravitons and neutrinos) unchanged. This occurs because the entropy of a gas of relativistic particles is proportional to the number of particles, which is invariant as the cosmos expands [44]. Therefore, if we assess the thermodynamic entropy inside the volume of the event horizon, the number of photons in that volume does not change. However, local observers embedded in their comoving horizon perceive a decrease in information (which gets more diluted), and therefore an increase in thermodynamic entropy. In sum, our theoretical account states that the cosmic expansion dictated by the cosmological constant of the quantum vacuum leads both to local decreases in information and increases in thermodynamic entropy in the spacetime accessible to local observers. A single observer detects the same macro-cosmic features everywhere and different observers detect the same macro-cosmic features (i.e., the Universe is homogeneous and isotropic), therefore it is feasible to extend our framework to every observer in the entire Universe.

## 5. Entangled Spacetime and Comoving Horizons: An Unexpected Link

We disregard quantum decoherence breaking due to afluctuating environment, working instead with the approximated hypothesis that quantum entanglement is maintained at infinite distance. In this context, our lack of knowledge of quantum issues in fluctuating environments in terms of objective and computable physical phenomena might be a great lack—also, for instance, in terms of pilot wave evolution in a noisy environment, in which the superposition of quantum states cannot be maintained and rebuilt on distances larger than the De Broglie length [45,46,47]. However, the recently-developed framework of quantum computational spacetime highlights the foremost role of entanglement entropy [5]. This leads us inside the spherical comoving horizon perceived by a local observer. Indeed, relationships do exist among the comoving horizon, quantum entanglement and information entropies. Recent claims suggest that quantum entanglement can be assessed in terms of opposite features on a four-dimensional hypersphere. It has been proposed that a separable state can be achieved for each of the entangled particles lying in S^2^, just by embedding them in a higher dimensional S^3^ space [48,49]. These authors view quantum entanglement as the simultaneous activation of signals in a 3D space mapped into a S^3^ hypersphere. The particles are entangled at the S^2^ level and un-entangled at the S^3^ hypersphere level, therefore a composite system is achieved, in which each local constituent is equipped with a pure state. It is noteworthy that the two issues of a comoving horizon and entanglement on a hypersphere are assessable through the framework described by the Borsuk–Ulam theorem, which states that “every continuous map $f:S^{n}\to R^{n}$ must identify a pair of antipodal points”—diametrically opposite points on an n-sphere [50,51].This means that at least some of the entropy values detected at opposite sides of the spherical comoving horizon display matching description, i.e., they are entangled.

In sum, in touch with the premises of quantum computational spacetime, a cosmic observer is merged inside a spherical comoving horizon that must necessarily display the entanglement entropy required by the theory.

## 6. Conclusions

Here we partially explain the occurrence of the second law of thermodynamics through the issue of the cosmic expansion, that leads to a diluted information for a local observer, and, consequently, to their detection of increases in thermodynamic entropy. The canonical approach is reverted: here we start from information, and reach the entropy, and not vice versa as generally assumed. It is noteworthy that information is also linked with the Shannon entropy, that holds for ergodic systems: The Universe is ergodic, because it is homogeneous and isotropic, at least at macroscales [52]. Our approach is based upon the relational formulations of quantum mechanics, where information is considered the most general paradigm to investigate cosmological and physical systems.

Therefore, (at least a part of) the increase in thermodynamic entropy might be correlated with cosmic expansion. Our hypothesis leads to (theoretically) testable previsions. When (and if) cosmic expansion decreases or relapses, the entropy perceived by local observers embedded in their comoving horizon must decrease, or even relapse. Furthermore, we predict that, due to cosmic dilation, the thermodynamic entropy detectable by local observers is increased in the current cosmic era, compared with previous periods.

Our suggestions are in touch with the results of [53]. These authors described how an observer located inside the Universe perceives time flow, while a hypothetical external observer perceives the Universe as motionless. According to [54], entanglement discloses time as an emergent phenomenon. By running their experiment in two different modes (“observer” and “super-observer” mode) they showed how the same energy-entangled Hamiltonian eigenstate can be perceived as evolving by the internal observers that test the correlations between a clock subsystem and the rest, whereas it is static for the super-observer. This means that, in touch with both our account and relational quantum mechanics, the description of cosmic quantities, such as thermal history or time passing, is meaningful in the framework of measurements performed by anobserver. The above-mentioned approximated hypothesis, i.e., that quantum entanglement is maintained at infinite distance (as it happens in the absence of environment in zero-noise quantum mechanics), can be improved by introducing the concept of “super-observer”, that replaces the non-existence of quantum decoupled observers. The observer is still considered as part of a quantum super-system, while the existence of a “super-observer” who watches the system, interacts with it, but does not form quantum super-super-systems is admitted. This framework comes out of the fact that the measure is basically a statistical (and hence classical and quantum-incoherent) output of interactions between systems embedded in a large-scale classical system. The observer is a classical system quantum-decoupled (by the noise) with the measured system: in the relational quantum mechanical approach, this property is attributed (implicitly) to the super-observer.

As stated above, the Cosmic Background Radiation points towards our Universe as strongly isotropic and homogeneous at cosmic macroscales. Inflationary expansion explains why the primeval 10^90^ causally-disconnected quantum “seeds” [31] led to the experimentally detected homogeneity and isotropy. Still, inflation would have amplified minute quantum fluctuations (pre-inflation) into slight density ripples of over- and under-density (post-inflation).Here the concept of hyperuniformity comes into play, i.e., the anomalous suppression of density fluctuations on large length scales occurring in amorphous cellular structures of ordered and disordered materials [55].The evolution of a given set of initial points takes place when, through Lloyd iterations, each point is replaced by the center mass of its Voronoi cell. This corresponds to a gradient descent algorithm which allows a progressive, general convergence to a random minimum in the potential energy surface. Klatt et al. [55] report that systems equipped with different initial configurations (such as, e.g., either hyper-fluctuating, or anisotropic, or relatively homogeneous point sets), converge towards the same high degree of uniformity after a relatively small number of Lloyd iterations (about 10^5^).This means that, in the systems’ final states, independent of the initial conditions, the cell volumes become uniform and the dimension less total energy converges towards values comparable to the deep local energy minima of the optimal lattice. Therefore, we are allowed to describe the cosmic evolution suddenly after the Big Bang in terms of Lloyd iterations, where the initial quantum seeds stand for initial point sets, progressively converted to point sets with a centroidal Voronoi diagram. In other words, the tiny perturbations in the primeval Universe which seed the later formation of cosmic macro-structures might stand for the starting points of the subsequent processes described in terms of Voronoi cells. This would permit observers to achieve, starting from countless different possible conformations of the primeval Universe, the detected isotropic and homogeneous Cosmic Background Radiation. Indeed, after just 10^5^ iterations, every possible initial system must converge towards an hyperuniform state, where observers perceive energy as very low and uniformity degree as very high.
